# Comparison of the probability of four anticonvulsant mood stabilizers to facilitate polycystic ovary syndrome in women with epilepsies or bipolar disorder—A systematic review and meta-analysis

**DOI:** 10.3389/fpsyt.2023.1128011

**Published:** 2023-05-09

**Authors:** Jing Guo, Yan Liu, Lingling Kong, Yaoyao Sun, Zhe Lu, Tianlan Lu, Haiying Qu, Weihua Yue

**Affiliations:** ^1^Peking University Sixth Hospital, Peking University Institute of Mental Health, Beijing, China; ^2^National Clinical Research Center for Mental Disorders (Peking University Sixth Hospital), Beijing, China; ^3^NHC Key Laboratory of Mental Health (Peking University), Beijing, China; ^4^Department of Psychology, Medical Humanities Research Center, Binzhou Medical University, Yantai, China; ^5^PKU-IDG/McGovern Institute for Brain Research, Peking University, Beijing, China; ^6^Chinese Institute for Brain Research, Beijing, China

**Keywords:** PCOS, mood stabilizers, valproate, carbamazepine, oxcarbazepine, lamotrigine

## Abstract

**Background:**

Patients treated with anticonvulsant mood stabilizers have a higher incidence of polycystic ovary syndrome (PCOS). However, there is no comparison between different anticonvulsant mood stabilizers. The purpose of this study was to systematically evaluate the prevalence of PCOS in women taking anticonvulsant mood stabilizers and compare the probability of PCOS caused by different anticonvulsant mood stabilizers.

**Methods:**

Five databases, namely PubMed, Embase, Web of Science, Cochrane Library, and Clinical Trials, were searched for literature on anticonvulsant mood stabilizers and PCOS published up to October 28, 2022. This meta-analysis was performed using Revman 5.4, Stata 14.0, and R4.1.0, and effect size pooling was performed in fixed- or random-effects models based on the results of *I*^2^ and Q-test, and the surface under the cumulative ranking curve (SUCRA) was used for analysis to assess the cumulative probability of drug-induced PCOS. Publication bias was assessed by funnel plot Egger's test and meta regression.

**Results:**

Twenty studies with a total of 1,524 patients were included in a single-arm analysis, which showed a combined effect size (95% CI) of 0.21 (0.15–0.28) for PCOS in patients taking anticonvulsant mood stabilizers. Nine controlled studies, including 500 patients taking medication and 457 healthy controls, were included in a meta-analysis, which showed OR = 3.23 and 95% CI = 2.19–4.76 for PCOS in women taking anticonvulsant mood stabilizers. Sixteen studies with a total of 1416 patients were included in a network meta-analysis involving four drugs, valproate (VPA), carbamazepine (CBZ), oxcarbazepine (OXC), and lamotrigine (LTG), and the results of the network meta-analysis showed that VPA (OR = 6.86, 95% CI = 2.92–24.07), CBZ (OR = 3.28, 95% CI = 0.99–12.64), OXC (OR = 4.30, 95% CI = 0.40–49.49), and LTG (OR = 1.99, 95% CI = 0.16–10.30), with cumulative probabilities ranked as VPA (90.1%), OXC (63.9%), CBZ (50.1%), and LTG (44.0%).

**Conclusion:**

The incidence of PCOS was higher in female patients treated with anticonvulsant mood stabilizers than in the healthy population, with VPA having the highest likelihood of causing PCOS. The most recommended medication when considering PCOS factors is LTG.

**Systematic review registration:**

identifier: CRD42022380927

## 1. Introduction

Anticonvulsant mood stabilizers are drugs that have certain therapeutic effects on epilepsy, bipolar disorder, and other diseases (1). At present, its definition remains highly controversial, but the consensus is that anticonvulsant mood stabilizers are a group of drugs with different pharmacological effects (2), which include antimanics (e.g., lithium carbonate), anticonvulsants (valproate, lamotrigine, carbamazepine, etc.), and atypical antipsychotics (quetiapine, amisulpride, aripiprazole, etc.), many of which are used primarily as antiepileptics. Common anticonvulsant mood stabilizers currently used in clinical practice include lithium, valproate (VPA), carbamazepine (CBZ), oxcarbazepine (OXC), lamotrigine (LTG), olanzapine, and others.

McEvoy et al. (3) showed that long-term use of anticonvulsant mood stabilizers, while providing therapeutic benefits, can also cause extrapyramidal adverse effects, lipid metabolism disorders, sex hormone changes, and other symptoms, which are more common in female patients (4). Menstrual disorder, amenorrhea, weight gain, polycystic ovary syndrome (PCOS), and so on caused by mood stabilizers will have a certain impact on patient's lives.

Polycystic ovary syndrome (PCOS) is a common endocrine disorder affecting 5%−10% of women of childbearing age (5, 6). The main clinical manifestations are irregular menstrual cycle, infertility, hirsuteness, and obesity (7). Research by Chittenden et al. (8) and Banning et al. (9) suggests that PCOS increases the risk of cardiovascular disease as well as the possibility of ovarian cancer. Talib et al. (10) and Zhuang et al. (11) showed an association between drugs and PCOS, which suggests that PCOS caused by drugs, especially PCOS caused by anticonvulsant mood stabilizers, deserves attention. Ernst and Goldberg (12) suggested that there are three theoretical explanations for drug-induced PCOS: the first explanation is that drug use will cause weight gain and obesity, which will lead to hyperandrogenemia and menstrual abnormalities; the second suggests that drug use can lead to increased levels of GABA aminobutyric acid, affecting hormone levels that trigger PCOS; the third explanation proposes that the drug prevents follicle maturation by inhibiting the conversion of testosterone to estradiol, ultimately leading to PCOS. Although there is some evidence that the use of anticonvulsant mood stabilizers is associated with the incidence of PCOS, few studies have compared the probability of different medications triggering PCOS.

At present, there are three diagnostic criteria for PCOS: the National Institutes of Health (NIH) criteria, the Homburg criteria, and Rotterdam criteria 2003. The NIH criteria were defined by the National Institutes of Health Consensus Conference, based on polycystic ovarian echograms and other functional disorders. The Homburg criteria were defined by Homburg, based on the diameter and number of two-dimensional ovarian planes. The diagnosis of Rotterdam criteria 2003 requires two of the three conditions: clinical manifestations, follicle size, and ovarian volume. Other endocrine causes (e.g., congenital adrenal hyperplasia, Cushing's syndrome, etc.) should be excluded for all diagnoses (13).

A meta-analysis by Hu et al. (14) pointed out that the incidence of PCOS in women treated with VPA was 1.95-fold higher than with other drugs. In recent years, studies by Zhang et al. (15) have shown that the use of VPA is associated with an increased incidence of PCOS. Some studies have also reported adverse reactions to anticonvulsant mood stabilizers such as CBZ and LTG, such as menstrual disorders, obesity, and abnormal reproductive hormones (12, 16). However, the current studies have all focused on the phenomenon of PCOS triggered by a particular drug, and few studies have compared the probability of PCOS triggered by different drugs to give a reference on the clinical use of anticonvulsant mood stabilizers.

To further explore the relationship between anticonvulsant mood stabilizers and PCOS in women, and to provide as comprehensive a reference as possible for the safe use of drugs, this analysis collected the literature on anticonvulsant mood stabilizers and PCOS published as of October 28, 2022, summarized, and merged the probability of anticonvulsant mood stabilizers causing PCOS. The aim was to evaluate the extent to which taking anticonvulsant mood stabilizers would induce PCOS compared with the healthy control group, and conduct network meta-analysis to rank four anticonvulsant mood stabilizers: VPA, CBZ, OXC, and LTG. The results are reported as follows.

## 2. Methods

A systematic review and network meta-analysis was conducted in accordance with the Preferred Reporting Items for Systematic Reviews and Meta-Analyses (PRISMA) guidelines. The protocol was registered with PROSPERO (CRD42022380927).

### 2.1. Search strategy and inclusion criteria

PubMed, Embase, Web of Science, Cochrane Library, and Clinical Trials were searched systematically for published literature in English up to 28 October 2022.

The search consisted of the following terms as Medical Subject Headings (MSH) and keywords appropriate to each database. The following search strategy was used: (“polycystic ovary syndrome” OR PCOS OR “polycystic ovaries”) AND (“anticonvulsant mood stabilizers” OR antimanic OR anticonvulsants OR “atypical antipsychotics” OR “lithium salts” OR “lithium carbonate” OR “propionate valproate” OR lamotrigine OR carbamazepine OR olanzapine OR quetiapine OR risperidone OR amisulpride). The search terms were adapted to the specific database, using a combination of subject terms such as MeSH (PubMed) and Emtree (EMBASE) and free terms.

According to the abbreviation PICOS, the selection criteria were as follows: Participants (P): we included patients who received a mood stabilizer treatment and excluded patients with major medical conditions (such as liver or kidney dysfunction in relation to cardiovascular disease or organic brain disorders) or with a history of substance use disorders. Interventions (I): take any mood stabilizer drug. Comparators (C): comparison with healthy population. Healthy controls were all without a significant history of epilepsy or other medical, neurological, or psychiatric disorders. Outcomes (O): number of people experiencing substance-induced PCOS (diagnosed using accepted diagnostic criteria). Study design (S): single-arm meta-analysis applied to single-group studies and case-control studies, control studies with healthy populations for meta-analysis of RCTs, and control studies taking anticonvulsant mood stabilizers for net meta-analysis.

### 2.2. Data collection and quality assessment

Two authors independently screened the article titles and abstracts, extracted the information, and assessed the quality. The base information of all included literature was extracted, including the name of the investigator, year, age of the subject, medication information, number of people in each group, and PCOS diagnostic criteria.

For the network meta-analysis, we applied the Cochrane Risk of Bias (ROB) tool to assess the risk of bias. The assessment included seven entries on random allocation scheme generation, allocation concealment, blinding of subjects and interventionists, blinding of outcome evaluators, incomplete outcome data, selective outcome reporting, and other biases. Each entry was assessed as low risk, unclear risk, and high risk (17, 18).

### 2.3. Data analysis

Single-arm meta-analysis were conducted based on Stata 14.0 software, meta-analyses of healthy controls were based on RevMan 5.4, and network meta-analyses were based on Stata 14.0 and R4.1.0 (19).

First, we performed a single-arm effect size pooling, which was used to examine the likelihood of taking any one mood stabilizer to cause PCOS. We used the Q-test to assess heterogeneity, and *P* < 0.1 was considered statistically significant. The *I*^2^ statistic was used to quantify heterogeneity, and *I*^2^ values closer to 0% indicated less heterogeneity and vice versa. Given the expected heterogeneity, we a priori used a random-effects model. When *I*^2^ < 50%, a fixed effects model was used. Combined effect size results greater than zero indicated that there was a likelihood of PCOS being triggered by taking anticonvulsant mood stabilizers. Funnel plots and Egger's tests were used to evaluate publication bias.

Second, we conducted a meta-analysis between anticonvulsant mood stabilizers and healthy people to examine the differences between anticonvulsant mood stabilizers use and non-use. The choice of random or fixed effects model was consistent with the one-arm study. Dichotomous variables were expressed using OR values and 95% CI. OR greater than one indicated a difference between use and non-use. Similarly, funnel plots and Egger's tests were used to assess publication bias.

Finally, we used network meta-analysis to assess the comparison of the likelihood of PCOS triggered by different anticonvulsant mood stabilizers. A healthy population was used as a reference for assessment. Heterogeneity was assessed using *I*^2^, which in turn led to the selection of a random or fixed model. Inconsistency between direct and indirect evidence was calculated using the nodal split method. The probability of different drugs causing PCOS was compared using the area under the cumulative ranking curve (SUCRA). The higher the SUCRA value, the more likely the drug induced PCOS. Using cumulative probability to predict the stability of results, the closer the cumulative probability result is to the cumulative ranking curve, the more stable the result is.

All statistical differences were considered significant when *P* < 0.05.

## 3. Results

### 3.1. Study selection and characteristics

**Figure 1:**
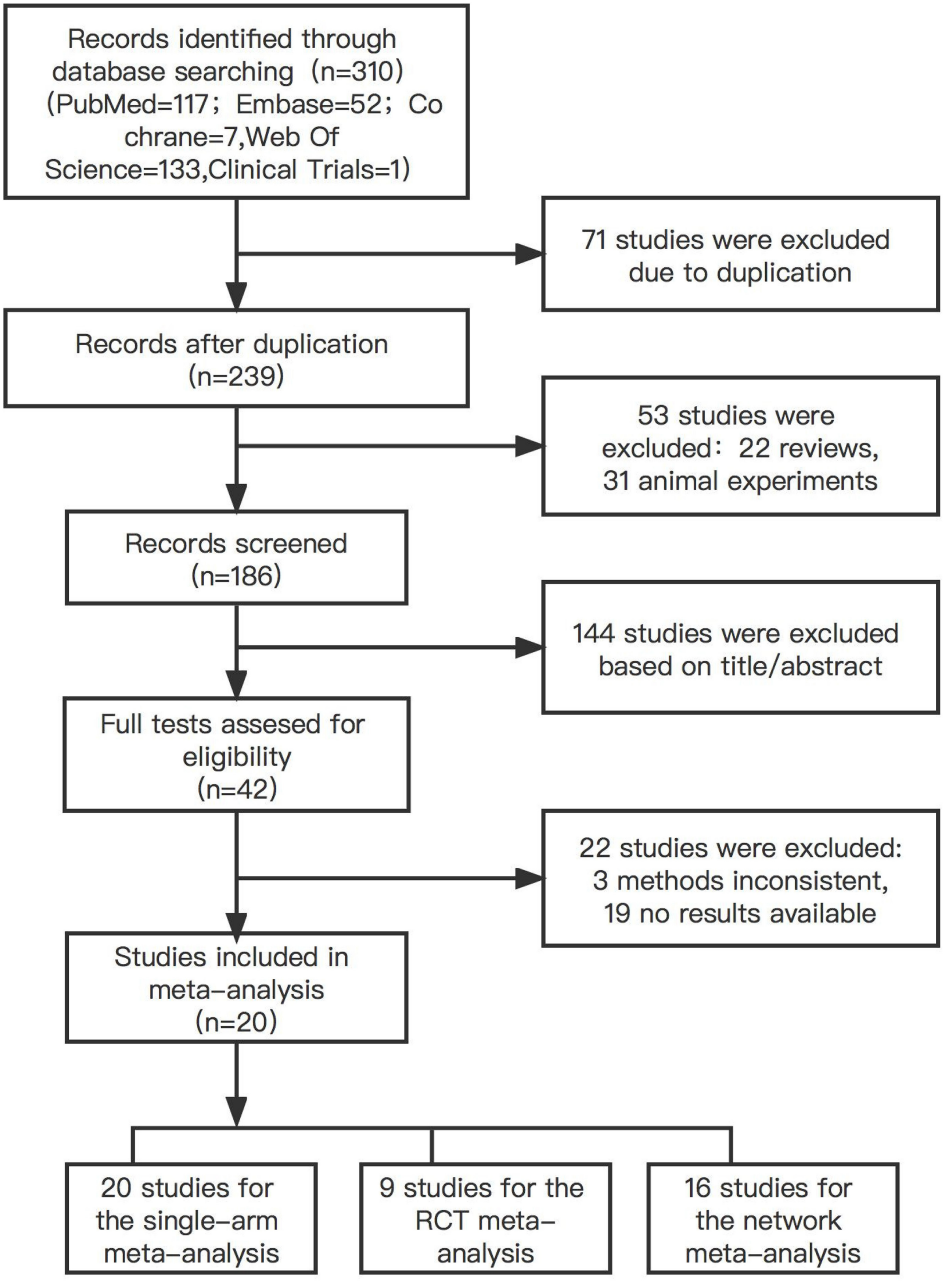
Flow chart of included studies. The figure described the route of literature screening. Among 310 researches retrieved, 20 studies were satisfied for the criteria of our study. Twenty studies were included in the single-arm meta-analysis, nine were included in the RCT meta-analysis, and 16 were included in the network meta-analysis.

A total of 185 papers were searched, with 71 duplicates, and 20 articles were found to have relevant information after screening. Figure 1 shows the flow chart for study selection.

Of these, 20 studies with a total of 1,524 patients were included in the single-arm analysis. In the RCT meta-analysis, nine controlled studies including 500 patients taking medication and 457 healthy controls were included in. Sixteen studies with a total of 1,416 patients were included in the network meta-analysis. Patients included in the study included both epilepsy and bipolar disorder patients (Table 1).

**Table 1:** List of included studies.

**Author (publication year)**	**Age (years)**	**Type of disease**	**Diagnostic tools**	**Group and sample size**	**Trial duration**	**References**
O'Donovan et al. (2002)	15–45	Bipolar disorder	NIH criteria^a^	VPA = 17 Health = 22	6 months	(20)
Löfgren et al. (2006)	18–40	Epilepsy	Homburg criteria^b^	CBA = 16 OXC = 19 Health = 36	NR	(21)
Löfgren et al. (2007)	18–40	Epilepsy	Homburg criteria^b^	VPA = 55 Mul = 93 Health = 170	NR	(22)
Luef et al. (2002)	16–40	Epilepsy	NIH criteria^a^	VPA = 22 Mul = 21	2 years	(23)
El-Khayat et al. (2004)	8-−18	Epilepsy	NIH criteria^a^	Mul = 44 Health = 40	6 months	(24)
Sidhu et al. (2018)	12–40	Epilepsy	Rotterdam criteria 2003^c^	VPA = 30 LTG = 27	12 months	(25)
Gorkemli et al. (2009)	17–39	Epilepsy	Rotterdam criteria 2003^c^	VPA = 40 Mul = 31	34 months	(26)
Bauer et al. (2000)	20–53	Epilepsy	NIH criteria^a^	VPA = 18 CBZ = 20	6 months	(27)
Mikkonen et al. (2004)	12.5–25.8	Epilepsy	Homburg criteria^b^	VPA = 7 Mul = 20 Health = 51	NR	(28)
Bilo et al. (2001)	16–42	Epilepsy	NIH criteria^a^	VPA = 13 Mul = 21 Health = 18	NR	(29)
Morrell et al. (2018)	13–70	Epilepsy	NIH criteria^a^	VPA = 80 LTG = 219	NR	(30)
Ayyagari et al. (2012)	13–45	Epilepsy	Rotterdam criteria 2003^c^	CBZ = 20 VPA = 20 Health = 20	6 months	(31)
Ogunjimi et al. (2020)	NR	Epilepsy	Rotterdam criteria 2003^c^	CBZ = 50 Health = 50	NR	(32)
Sahota et al. (2008)	14–45	Epilepsy	NIH criteria^a^	VPA = 30 CBZ = 10	6 months	(33)
Rasgon et al. (2005)	18–45	Bipolar disorder	NIH criteria^a^	VPA = 50 Mul = 22	NR	(34)
Betts et al. (2003)	NR	Epilepsy	NIH criteria^a^	VPA = 54 Mul = 51 Health = 50	NR	(35)

a NIH criteria: National Institutes of Health (NIH) consensus conference definition, echogram of polycystic ovaries, ovulatory dysfunction (polymenorrhoea, amenorrhea, or oligomenorrhoea), clinical and/or biochemical evidence of hyperandrogenism and exclusion of other endocrine disorders (e.g., hyperprolactinemia, Cushing's syndrome, congenital adrenocortical hyperplasia, etc.).

b Homburg criteria: The criteria of PCOS were defined by Homburg. The ovaries were considered to be polycystic if eight or more subcapsular follicles of 2–8 mm in diameter in one two-dimensional plane were detected in either of the ovaries.

c Rotterdam criteria 2003: Two of the three following criteria were fulfilled: (1) sparse or anovulation, (2) Clinical manifestations of hyperandrogen and/or hyperandrogenemia, (3) Polycystic ovarian changes: ≥12 follicles with a diameter of 2–9 mm in one or both ovaries, and/or ovarian volume ≥10 ml. In addition, other hyperandrogenic causes, such as congenital adrenal hyperplasia, Cushing's syndrome, and androgen-secreting tumor, were excluded.

### 3.2. Quality assessment

The quality of studies included in the network meta-analysis was assessed according to the risk of bias assessment tool provided by the Cochrane Handbook. For the random sequence generation, three studies described the specific random allocation scheme and were rated as “low risk,” while the remaining 13 studies did not describe it and were rated as “unclear.” For the allocation concealment, one study was assigned according to certain criteria and was rated as “high risk,” one study mentioned allocation concealment and was rated as “low risk,” and the remaining 14 studies were rated as “unclear risk.” Low risk of bias for blinding of participants and personnel, blinding of outcome assessment, incomplete outcome data, and selective reporting (Supplementary Figure 1).

### 3.3. Single-arm meta-analysis

**Figure 2:**
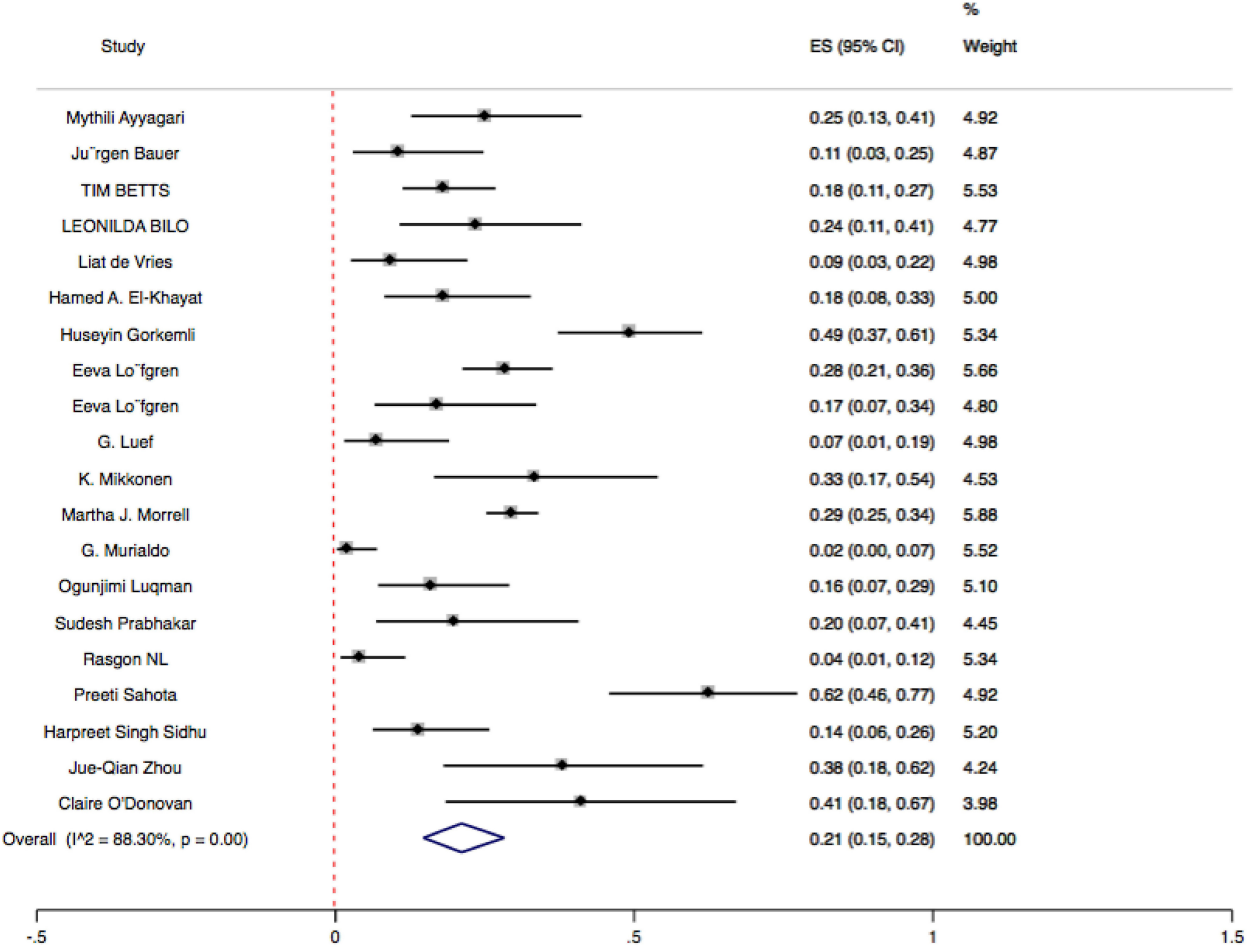
Single-armed forest plot. Single-armed meta-analysis forest plot. Random-effects model was used to combine data from 20 studies on mood stabilizers. The size of the gray plot proportional to weight in meta-analysis. Black lines, show confidence intervals.

Twenty studies (20–39) were quite heterogeneous (*I*^2^ = 88.30% > 50%, *P* < 0.1); the results of the sensitivity analysis showed that three studies (Martha, G. Murialdo, Huseyin) had a large effect on heterogeneity, and after removing these the remaining 17 still had a large heterogeneity (*I*^2^ = 80.04% > 50%, *P* < 0.1). Therefore, the random effects model was used to analyze the 20 studies. The combined effect value (ES) of 20 studies was 0.21 (95% CI = 0.15–0.28). The results were statistically significant (*Z* = 9.97, *P* < 0.05). This is detailed in the forest plot below (Figure 2).

The presence of publication bias in this study was examined by plotting a funnel plot. The funnel plot (Supplementary Figure 2) and Egger's test results (*P* = 0.626 > 0.05) indicate the funnel plot is symmetric and there was no statistically significant publication bias in the current study.

### 3.4. RCT meta-analysis

Nine papers (20–22, 24, 28, 29, 31, 32, 35) were included in this study. The heterogeneity test showed that *I*^2^ = 9% < 50%, *P* = 0.36 > 0.1, indicating that the heterogeneity among the selected literature in this study was not statistically significant, and fixed effects could be selected for meta-analysis.

**Figure 3:**
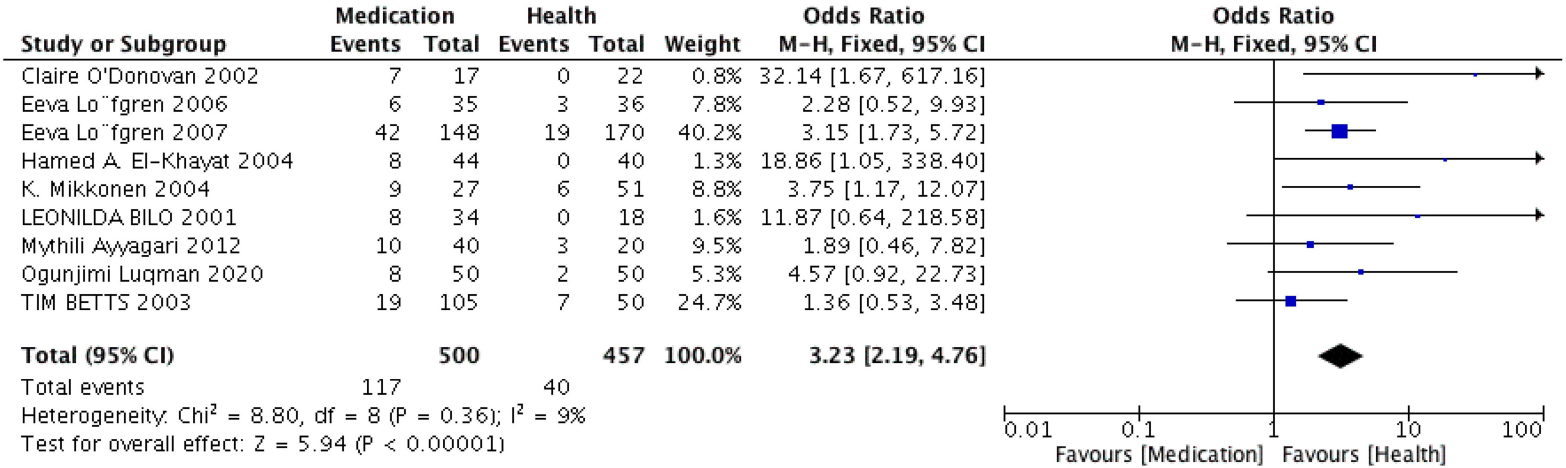
Single-armed forest plot. Single-armed meta-analysis forest plot. Random-effects model was used to combine data from 20 studies on mood stabilizers. The size of the gray plot proportional to weight in meta-analysis. Black lines, show confidence intervals.

Based on the fixed effects model, the combined results of nine healthy controlled studies suggested that anticonvulsant mood stabilizers could cause PCOS (OR = 3.23, 95% CI = 2.19–4.76), and the results were statistically significant (*Z* = 5.94, *P* < 0.05). This is shown in Figure 3. Egger's test results (*P* = 0.111 > 0.05) indicate there was no statistically significant level of publication bias.

### 3.5. Network meta-analysis

#### 3.5.1. Evidence of relationship

**Figure 4:**
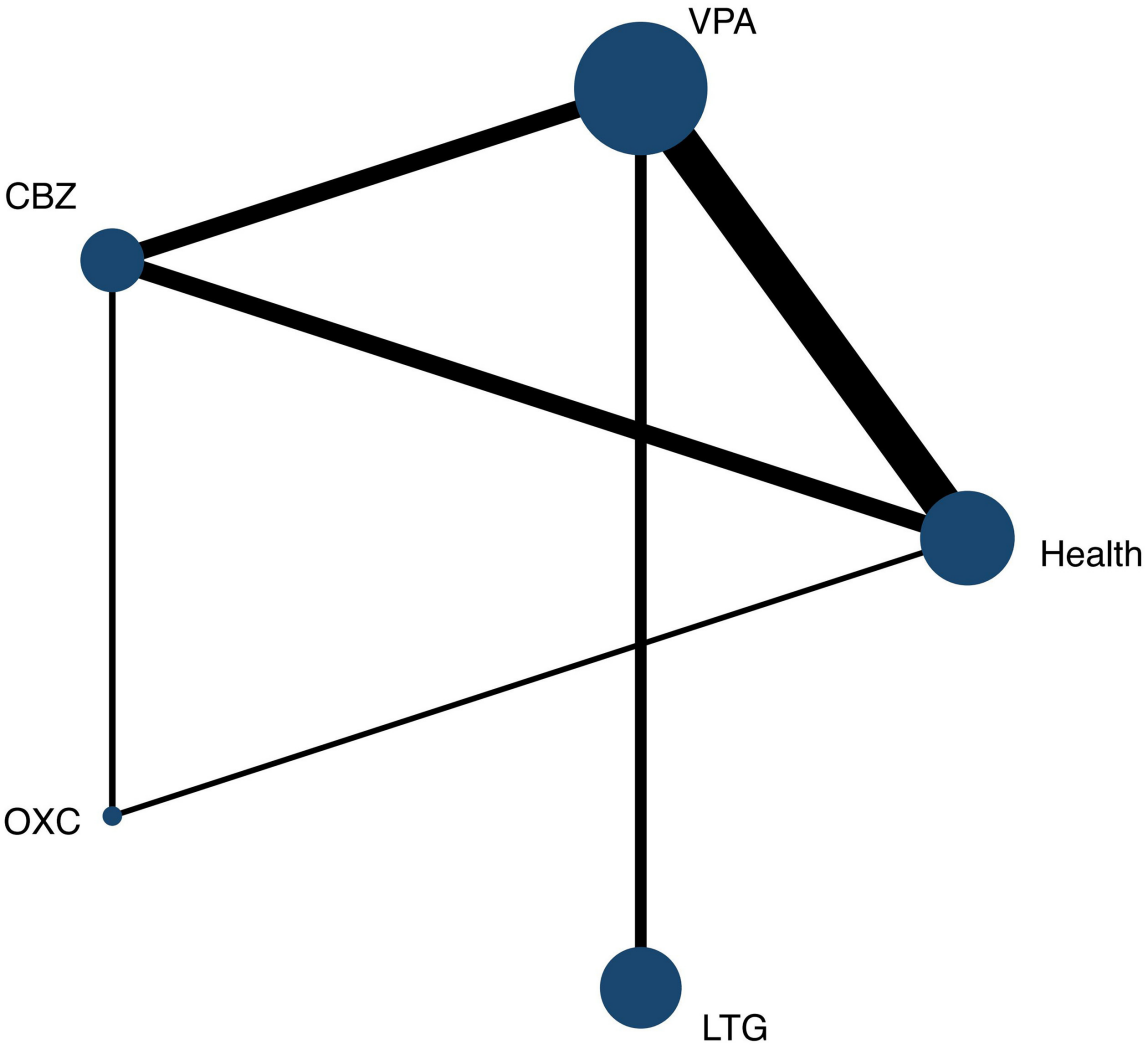
Network evidence plot for PCOS. VPA, valproate; CBZ, carbamazepine; OXC, oxcarbazepine; LTG, lamotrigine; Health, healthy control. One node represents an intervention and larger nodes represent more studies. The lines between nodes represent direct comparisons. The larger the connection line, the larger the number of direct comparison documents.

A total of six direct comparisons and four indirect comparisons existed for the 16 included studies (20–35). The relationship of evidence for all outcome indicators is shown in Figure 4. A node in the evidence diagram represents an intervention and the size of the node represents the number of studies that included that intervention. The presence of connecting lines between nodes indicates the presence of evidence for direct comparisons, and the absence of connecting lines indicates the absence of evidence for direct comparisons. The thickness of the connecting line represents the amount of literature included between the two interventions, with thicker lines representing more literature included.

#### 3.5.2. Heterogeneity and inconsistency tests

The overall heterogeneity *I*^2^ value for the study outcome indicators was 12%, suggesting no significant heterogeneity. The results of the inconsistency test for the two closed loops (Health—CBZ—OXC, Health—VPA—CBZ) showed that the inconsistency factor IF ranged from 0.48 to 0.50, indicating good consistency across the closed loops (Supplementary Figure 3). The results of the nodal cut test showed no statistically significant differences between the groups (*P* > 0.05).

#### 3.5.3. Comparison of four anticonvulsant mood stabilizers

A total of four drugs, VPA, CBZ, OXC, and LTG, were included. The results of the network meta-analysis showed that VPA (OR = 6.86, 95% CI = 2.92–24.07), CBZ (OR = 3.28, 95% CI = 0.99–12.64), OXC (OR = 4.30, 95% CI = 0.40–49.49), and LTG (OR = 1.99, 95% CI = 0.16–10.30). These drugs were significantly more likely to cause PCOS than the healthy population (all *P* < 0.05); see Supplementary Figure 4.

**Figure 5:**
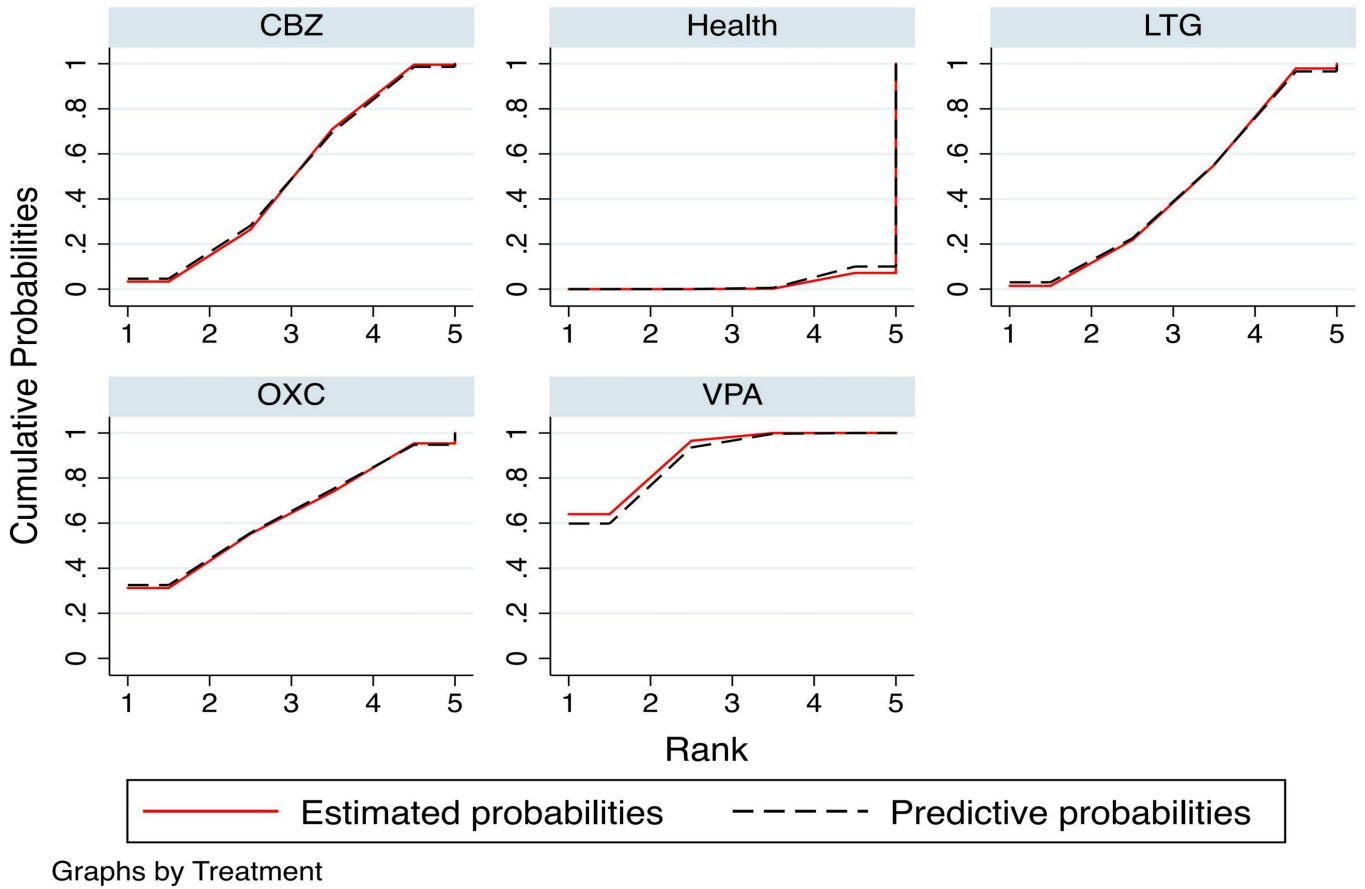
Cumulative probability ranking and prediction. Red solid line: estimated probabilities; Black dotted line: predictive probabilities.

The SUCRA range is between 0 and 100; the closer the value is to 100, the higher the ranking of the drug and the greater the probability of triggering PCOS. The SUCRA ranking result is VPA (90.1%), OXC (63.9%), CBZ (50.1%), and LTG (44.0%). The predicted results for the cumulative probabilities showed general agreement with the above results, with good stability of the results. The specific results are shown in Figure 5.

## 4. Discussion

Results based on a random effects model combining effect sizes from 20 studies showed that anticonvulsant mood stabilizers' use had an impact on the prevalence of PCOS in women (ES = 0.21, 95% CI = 0.15–0.28). Results from a meta-analysis of nine studies based on a fixed effects model showed that women taking anticonvulsant mood stabilizers were more likely to be diagnosed with PCOS compared to healthy controls (OR = 3.23, 95% CI = 2.19–4.76). Using the healthy control population as a reference to assess the likelihood of PCOS with the four anticonvulsant mood stabilizers, the network meta-analysis showed that the most likely of the four drugs to cause PCOS was VPA (OR = 6.86, 95% CI = 2.92–24.07) and the safest drug was LTG (OR = 1.99, 95% CI = 0.16–10.30), with OR results consistent with the SUCRA results: VPA (90.1%), OXC (63.9%), CBZ (50.1%), and LTG (44.0%).

Of the 20 studies we included in our net meta-analysis, 13 (20, 22, 23, 25–31, 33–35) involved the use of VPA. VPA (valproate, valproic acid, 2-propylpentanoic acid) is a simple branched-chain carboxylic acid that is a common antiepileptic drug and a common mood stabilizer used to treat bipolar disorder (40, 41). Verrotti et al. (42) suggest that VPA may disrupt ovarian function and androgen synthesis by acting on the hypothalamic-pituitary-ovarian axis. Isojärvi et al. (43–45) have shown in a series of studies that VPA increases the incidence of polycystic ovaries and PCOS. In addition, studies by Atif et al. and Okanović et al. also indicate a higher risk and diagnosis rate of PCOS among women taking VPA (46–48). The prevalence of PCOS in the general population is 5%−10%, and the risk of taking VPA is 1.95 times greater than not (5, 6, 10). Our results are consistent with these studies. All these results suggest to us that VPA is a very frequently used drug in clinical practice and has a higher risk of causing PCOS compared to other drugs.

Another phenomenon of interest and discussion in the choice of clinical medication is that Joffe et al. found that PCOS caused by VPA resolved in three quarters of patients after discontinuation of the medication through a follow-up of patients (49). Further observational trials are needed to determine whether VPA-induced PCOS is temporary and whether it can resolve on its own after discontinuation.

OXC can be used as a monotherapy or adjunctive treatment for partial epilepsy and is also indicated for affective-psychotic disorders (21, 50). The study by Luef et al. (51) noted that OXC stimulates GnRH neurons and that the released GnRH promotes the secretion and release of large amounts of luteinizing hormones, follicle-stimulating hormones, and testosterone from the pituitary gland and testes, and that the incidence of polycystic ovaries in women treated with OXC is as high as 60%. The results of this study showed that OXC was second only to VPA in terms of the probability of PCOS, with an OR of 4.46 (95% CI = 0.47–42.34) between OXC and PCOS, which is consistent with the results of Luef et al. Thus, OXC is not a preferred mood stabilizer when PCOS is taken into account.

A total of five of the papers included in this network meta-analysis were associated with CBZ (20, 27, 31–33). CBZ is structurally related to tricyclic antidepressants and has been used as an antiepileptic drug (52) since 1965, and later also as a mood stabilizer in the treatment of bipolar disorder and in the treatment of trigeminal neuralgia (53). Sahota et al. (33), Bilo et al. (54), and other studies have reported menstrual abnormalities, polycystic ovaries, and PCOS associated with CBZ use, and the findings suggest that CBZ is less likely to trigger side effects compared to VPA. These findings are consistent with the results of this meta-analysis.

LTG is an anticonvulsant drug that is also approved for the maintenance treatment of bipolar depression with bipolar disorder (55). Our findings show that LTG has a better safety profile compared to other anticonvulsant mood stabilizers. This is consistent with the findings of Li et al. (16).

Sidhu et al. (56) showed that genetic predisposition, environmental factors, and weight gain and insulin resistance are important factors in the development of PCOS. Nestleret et al. (57) also showed that up to 40% of all PCOS is caused by obesity, in line with findings from Reynold et al. (58). However, weight gain is one of the common side effects of psychiatric drugs (59, 60). This suggests to us that patients should be monitored for changes in their weight while on medication, and if there is significant weight gain, it is recommended that treatment regimens are adjusted and medication changed in a timely manner so that PCOS can be better prevented.

The study by Betts et al. (35) also noted that age had an effect on antipsychotic-induced PCOS: the probability of PCOS being triggered by antipsychotics was higher in patients under 25 years of age (52% for VPA and 35% for LTG and CBZ) than over 25 years of age (37% for VPA and 1% for LTG and CBZ). The study by Morrell et al. (30) also noted that antipsychotic-induced PCOS problems were more relevant in women under 25 years of age compared to those over 25 years of age. The studies all concluded similarly to Isojärvi et al. (43): PCO was present in 60% of women using VPA before the age of 20 years. However, previous literature suggests that more caution should be exercised in the use of medication in patients aged 20–25 years with psychiatric disorders, with the addition of polycystic-related monitoring if necessary, and that for women patients of childbearing age, a trade-off between treatment efficacy and metabolic health, reproductive status, and adverse drug reactions needs to be made for optimal use (61).

In the 16 studies included in this review, no confirmed diagnosis of PCOS according to age was reported, therefore, it was not necessary to perform a subgroup analysis of the included subjects according to age grouping. Further analysis can be carried out next by incorporating the age factor.

In addition, because PCOS can have a serious impact on the patient's quality of life, it may lead to a worsening of the patient's emotional problems (62, 63). The physical symptoms of PCOS can also cause psychological stress, leading to worries about fertility and health problems, which can affect patients' compliance with treatment (63). The emphasis on adequate dosage and treatment requires patients to take medication for long periods, and the risks associated with long-term medication require careful weighing of medication choices, careful attention during the course of medication, and prompt adjustment when problems are identified. This is particularly important for women with mental illness.

In summary, the use of anticonvulsant mood stabilizers increases the risk of PCOS in women. VPA is more likely to trigger PCOS than other drugs, and VPA needs to be used with more caution, especially in women under the age of 25. More research is needed to test whether patients can recover after VPA is discontinued. LTG has the least effect on PCOS compared to other medications and may be considered as a priority when PCOS is included as a consideration. In addition, clinicians and caregivers need to be aware of the possible side effects associated with anticonvulsant mood stabilizers, aware of possible weight gain, menstrual disorders, etc., and adjust the treatment plan if necessary to maximize drug effectiveness and medication safety.

## Data availability statement

The original contributions presented in the study are included in the article/Supplementary material, further inquiries can be directed to the corresponding authors.

## Author contributions

WY and JG conceived and designed the study. JG, YL, YS, and ZL extracted the data and assessed the risk of bias. JG and YL analyzed the data. WY supervised the data analyses. JG, LK, YS, ZL, TL, and HQ interpreted the data. JG drafted the manuscript, LK, WY, TL, and HQ contributed to revising the manuscript. All authors had full access to all the data in the study and had final responsibility for the decision to submit for publication.
