# Supplementary material for: Comparison of the probability of four anticonvulsant mood stabilizers to facilitate polycystic ovary syndrome in women with epilepsies or bipolar disorder—A systematic review and meta-analysis
Source: Front Psychiatry. 2023 May 9;14:1128011. doi: 10.3389/fpsyt.2023.1128011 (PMC10203219; doi:10.3389/fpsyt.2023.1128011)
Supplement: Supplementary file 1 [file Data_Sheet_1.PDF]

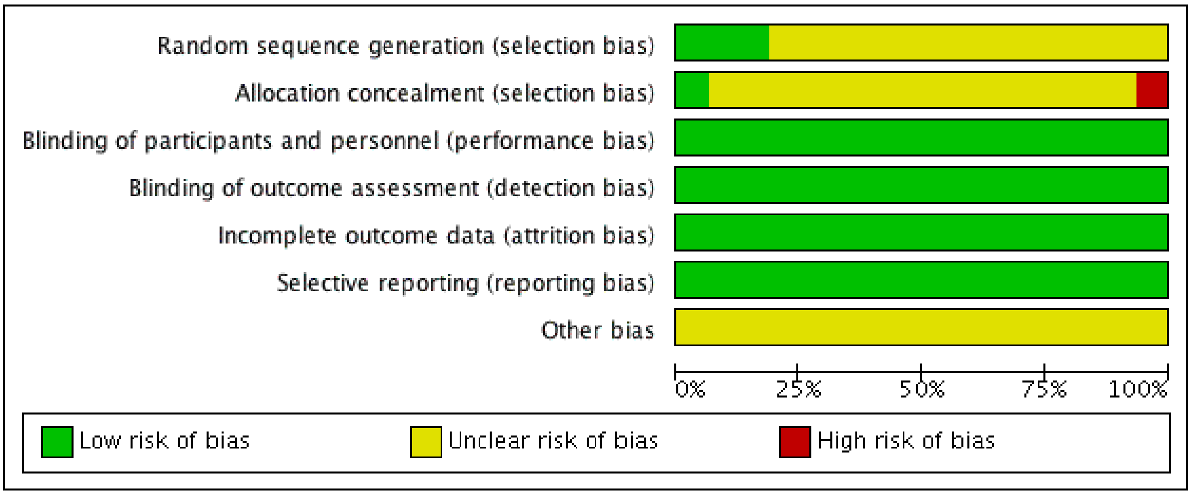

Supplementary 1 Quality assessment. The included studies were evaluated for seven items: random sequence generation, assignment concealment, blind researchers and subjects, blind outcome evaluation, integrity of outcome data, selective reporting of research results, and other bias. Green means low risk of bias, yellow means unknown risk of bias, and red means high risk of bias.

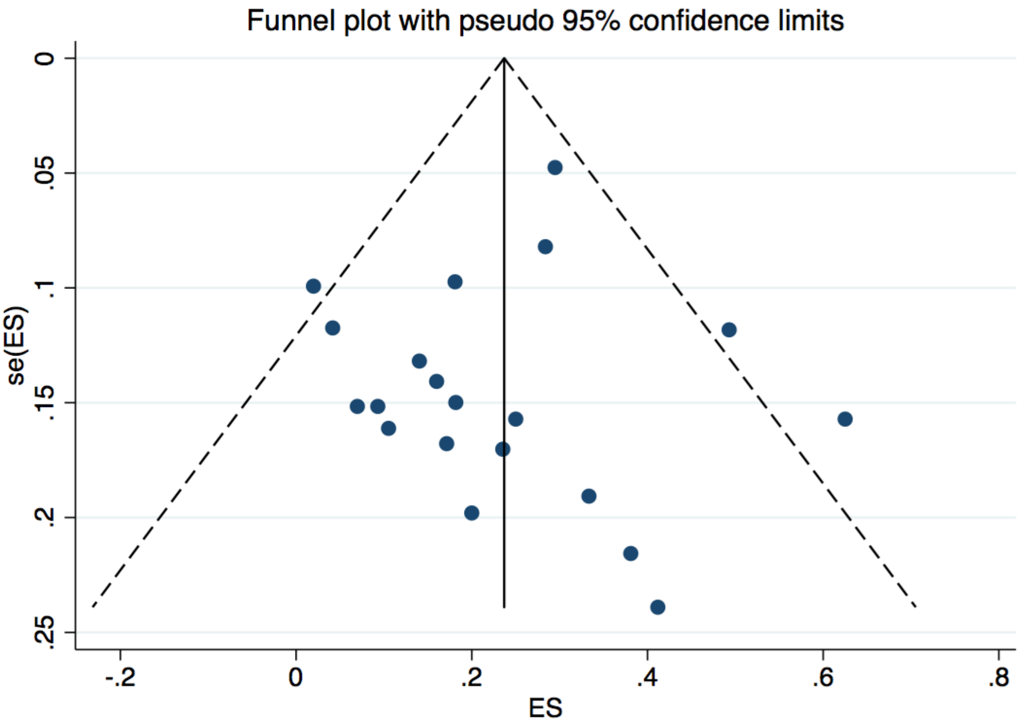

Supplementary 2 Bias test funnel plot. The two dashed lines represent 95% confidence intervals and the funnel plot is essentially symmetrical and within the confidence interval, representing the absence of publication bias in this study.

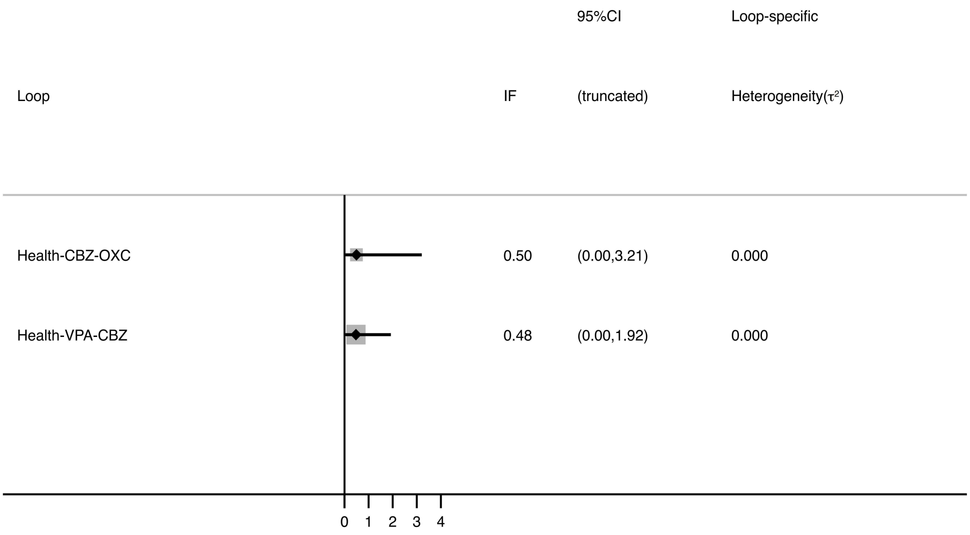

Supplementary 3 Loop inconsistency test. There were three closed loops in this study and the inconsistency test showed good agreement between the loops.

VPA				
0.45 (0.01, 6.69)	CBZ			
1.31 (0.12, 14.42)	0.6 (0.04, 3.55)	OXC		
0.48 (0.12, 1.56)	0.62 (0.05, 7.04)	0.29 (0.02, 1.01)	LTG	
6.86 (2.92, 24.07)	3.28 (0.99, 12.64)	4.3 (0.4, 49.49)	1.99 (0.16, 10.3)	Health

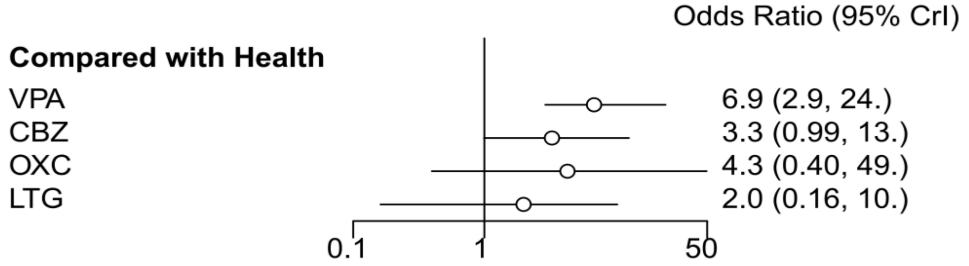

Supplementary 4 Inverted triangle and forest plot compared to healthy population. OR is the ratio of ratios, CI is the confidence interval. Comparisons between groups. an OR greater than 1 indicates that drug-induced PCOS is higher than in the healthy population.
